# Books Received

**Published:** 1867-12

**Authors:** 


### BOOKS RECEIVED.

*Report on Meteorology, Medical Topography, and Epidemic Diseases of Illinois.* By R. C. HAMILL, M.D., Chicago, Ill.

A valuable contribution to the Transactions of the American Medical Association, from which the practitioners of Illinois, especially, cannot fail to gather many useful hints.

*Catalogue of the Surgical Section of the United States Army Medical Museum.* Prepared under the direction of the Surgeon-General, U.S. Army. By ALFRED A. WOODHULL, Assistant-Surgeon and Brevet-Major, U.S.A. Washington: Government Printing Office. 1867.

We are under great obligations to the Department, for a copy of this most important volume. It does great credit to the industry and zeal of the Medical Staff of the Army. A more extended notice and analysis of its contents will appear in an early No. of the JOURNAL.

*A Contribution to the History of the Hip-Joint Operations Performed during the Late Civil War:* Being the Statistics of Twenty Cases of Amputations and Thirteen of Resections, at this Articulation, in the Southern Service. By PAUL F. EVE, M.D., Prof. of Surgery in the University of Nashville, Tenn.

*The Gospel Among the Animals, or Christ with the Cattle.* By SAMUEL OSGOOD, D.D. New York: Samuel R. Wells, Publisher, 389 Broadway. 1867.

*A Complete List of the Muscles of the Human Body.* A Chart —Size 24x38 inches. By W. LITTLE, M.D., Chicago, Ill.

A convenient map for students and practitioners, showing at a glance, the muscles of the body, their attachments, and uses.

*Lectures on the Diseases of Women.* By CHARLES WEST, M.D., F.R.C.P., Examiner in Midwifery at the University of London, etc. Third American, from the Third and Revised English Edition. Philadelphia: Henry C. Lea. 1867.

It is unnecessary to introduce this work to medical readers, as it has already passed among the standard treatises which should be found in every professional library. In the present edition, we notice valuable revisions and additions, suggested by the author's larger experience and study. The subjects, Uterine Hæmatocele and Ovarian Disease, have been extensively elaborated.

*Pope's Essay on Man.* By ALEXANDER POPE, with notes by S. R. WELLS. Beautifully Illustrated. S. R. Wells Publisher, 389 Broadway, New York. 1867.

*Then and Now:* A Discourse Introductory to the Forty-Third Course of Lectures in the Jefferson Medical College of Philadelphia. By S. D. GROSS, M.D., Prof. of Principles and Practice of Surgery.

*The Principles of Medicine,* By JOHN M. SCUDDER, M.D., Professor of the Principles and Practice of Medicine in the Eclectic Medical Institute of Cincinnati, Ohio; Author of a "Treatise on the Diseases of Women," etc. Cincinnati: 1867. From the Author.

This work contains, in addition to much that, of course, is found in standard treatises on the subject, an outline of the supposed peculiar views of the so-called "Eclectics." We fail to see the necessity of any such prefix, or the real importance of the suggested differences. Scientific medical men may be fairly supposed to practice in accordance with the principles of science and not the mere dogmas of a sect.

*The Physician's Hand-Book for 1868.* By WILLIAM ELMER, M.D. New York: W. A. Townshend & Adams, Publishers, No. 434 Broome Street.

The receipt of this well-known hand-book and diary should have been earlier acknowledged—but, "good wine needs no bush."

*Seventeenth Anniversary Meeting of the Illinois State Medical Society.* Held in Springfield, June 4 and 5, 1867. Robert Fergus' Sons, Printers, 12 & 14 Clark St. 1867. Pp. 212.

*Rand's Medical Chemistry,* the publication of which, by T. Elwood Zell & Co., was noticed in a previous number of the

JOURNAL, we find, on examination, an exceedingly convenient and useful little work. Although designed mainly for students, it is well worth perusal or reference by practitioners. We cordially commend it to our readers.

*The Atlantic Monthly*, December, 1867.—Ticknor & Fields still sustain the high reputation achieved by this literary magazine. It is, unquestionably, the leading monthly of the country for family reading. In the ensuing volume, its readers are promised a new novel by Dickens, which announcement alone must largely increase its subscription list.

*Our Young Folks*, published by the same house, is the best magazine for younger readers with which we are acquainted. *Our* young folks delight in its contents, and the old folks while away many a pleasant hour in discussing its contents.

*Every Saturday*, continues fruitful in good reading, and, during the last year, has afforded many articles of a high order of ability.

*Southern Journal of Medical Sciences.*—The November No. of this excellent quarterly is at hand, filled with valuable and interesting matter. It is announced that arrangements have been made, whereby it is hereafter to be practically consolidated with the *New Orleans Medical and Surgical Journal*, and the talent exercised in the support of the two is hereafter to be combined. This seems to us a wise movement. $6.00 *per annum*, in advance. Address Dr. W. S. MITCHELL, Lock Box 890, P.O., New Orleans.

